# An In Vivo Assessment of the Effect of Hexane Extract from *Endlicheria paniculata* Branches and Its Main Compound, Methyldehydrodieugenol B, on Murine Sponge-Induced Inflammation

**DOI:** 10.3390/molecules28135247

**Published:** 2023-07-06

**Authors:** Bruno Antonio Ferreira, Rafael Aparecido Carvalho Souza, Francyelle Borges Rosa de Moura, Tiara da Costa Silva, Tais da Silva Adriano, Eduardo de Faria Franca, Raquel Maria Ferreira de Sousa, Fernanda de Assis Araújo, João Henrique Ghilardi Lago, Alberto de Oliveira

**Affiliations:** 1 Department of Physiological Sciences, Federal University of Uberlandia, Uberlandia 38408-100, Brazil; bruno.antonioferreira70@gmail.com (B.A.F.); folaraujo@gmail.com (F.d.A.A.); 2 Center for Natural and Human Sciences, Federal University of ABC, Santo Andre 09210-180, Brazil; 3 Institute of Chemistry, Federal University of Uberlandia, Uberlandia 38408-100, Brazil; rafasouza27@ufu.br (R.A.C.S.); tiaracosta@ufu.br (T.d.C.S.); taisadriano9@gmail.com (T.d.S.A.); eduardofranca@ufu.br (E.d.F.F.); rsousa@ufu.br (R.M.F.d.S.); 4 Department of Cell Biology, Histology and Embryology, Federal University of Uberlandia, Uberlandia 38408-100, Brazil; francyelle.moura@ufcat.edu.br; 5 Department of Biological Sciences, Federal University of Catalao, Catalao 75704-020, Brazil

**Keywords:** antifibrogenic, antiangiogenic, pharmacokinetic parameters

## Abstract

The present study aims to explore the anti-inflammatory potential activity of the hexane extract from branches (HEB) of *Endlicheria paniculata* (Lauraceae) and its main compound, methyldehydrodieugenol B, in the inflammatory response induced by a murine implant sponge model. HPLC-ESI/MS analysis of HEB led to the identification of six chemically related neolignans, with methyldehydrodieugenol B as the main compound. An in silico analysis of the pharmacokinetic parameters of the identified compounds suggested moderate solubility but good absorption and biodistribution in vivo. Thus, the treatment of mice with HEB using in vivo assays indicated that HEB promoted pro-inflammatory, antiangiogenic, and antifibrogenic effects, whereas treatment with methyldehydrodieugenol B caused anti-inflammatory, antifibrogenic, and antiangiogenic effects. The obtained results shown the therapeutic potential of HEB and methyldehydrodieugenol B in the treatment of pathologies associated with inflammation and angiogenesis, including chronic wounds.

## 1. Introduction

Inflammation is an essential defense mechanism of the body, as it is a way of signaling for the immune system to heal and solve a problem, such as cell damage and infections caused by viruses and bacteria [1]. However, when the inflammation process lasts for a long time, chronic inflammation can lead to several health problems, such as rheumatoid arthritis, systemic lupus erythematosus, multiple sclerosis, and many other debilitating diseases throughout life, which also cause increased mortality and high costs for therapy and care [2,3,4]. Among the various drugs used to treat inflammatory diseases, the phenylpropanoid class is one of the most significant. For example, 14 of the 53 anti-inflammatory drugs that were approved over nearly four decades are derived from or based on this chemical class, such as suprofen, flunoxaprofen, and butibufen [5]. In addition, studies have also demonstrated the anti-inflammatory effects of phenylpropanoid treatment using in vivo models, including dehydrodieugenol B [6], anethole [7], and ethyl *p*-coumarate [8]. Additionally, studies have shown the anti-inflammatory activity of various plant extracts, such as ginger [9] and curcuma [10].

Previous studies have shown that the hexane extract from the leaves of *Endlicheria paniculata* and dehydrodieugenol B, a neolignan isolated as the main constituent of the studied extract, exhibited an anti-inflammatory effect [6]. As part of our continuous studies, in the present work, the hexane extract from branches (HEB) of *E. paniculata* was chemically analyzed, allowing for the identification of six structurally related neolignans. Each compound was subjected to in silico analysis aimed at determining some of its pharmacokinetic/physicochemical properties and drug-likeness parameters. Based on these results, HEB and methyldehydrodieugenol B were selected for the evaluation of in vivo anti-inflammatory activity.

## 2. Results and Discussion

### 2.1. Chemical Analysis

**Figure 1:**
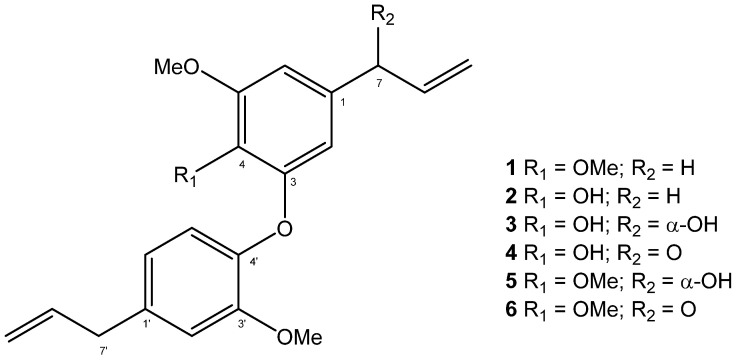
Chemical structures of Compounds **1**–**6,** identified on HEB from *E. paniculata*.

HPLC-ESI/MS analysis of HEB (Figure S10) allowed for the identification of methyldehydrodieugenol B (**1**), dehydrodieugenol B (**2**), 1-[(7*R*)-hydroxy-8-propenyl]-3-[3′-methoxy-1′-(8′-propenyl)phenoxy]-4-hydroxy-5-methoxybenzene (**3**), 1-(7-oxo-8-propenyl)-3-[3′-methoxy-1′-(8′-propenyl)phenoxy]-4-hydroxy-5-methoxybenzene (**4**), 1-[(7*R*)-hydroxy-8-propenyl]-3-[3′-methoxy-1′-(8′-propenyl)phenoxy]-4,5-methoxybenzene (**5**), and 1-(7-oxo-8-propenyl)-3-[3′-methoxy-1′-(8′-propenyl)phenoxy]-4,5-dimethoxybenzene (**6**) [11,12,13], as shown in Figure 1. Despite the occurrence of Compounds **1**–**3** in leaves of *E. paniculata* [6], this is the first report of Compounds **4**–**6** in an *Endlicheria* species (Table S1).

Additionally, methyldehydrodieugenol B (**1**), the main compound found in HEB, was isolated using different chromatographic steps. Its characterization was performed through a comparison of its ^1^H, ^13^C, DEPT, HSQC, and HMBC NMR data (Figures S1–S8 and Table S2) with previously reported figures [12]. The NMR spectra of Compound **1** was virtually identical to that of dehydrodieugenol B (**2**) [6], but it displayed extra peaks at δ_H_ 3.88 and δ_C_ 61.2 on its ^1^H and ^13^C NMR spectra, respectively. This was attributed to the additional methoxy group on C-4 [12], which was confirmed by analysis of ESI-HRMS data due to the presence of a peak at *m/z* 363.1585 [M + Na]^+^, in accordance with the molecular formula of Compound **1**: C_21_H_24_O_4_.

### 2.2. In Silico Analysis of the Pharmacokinetic Parameters of Compounds 1–6

**Figure 2:**
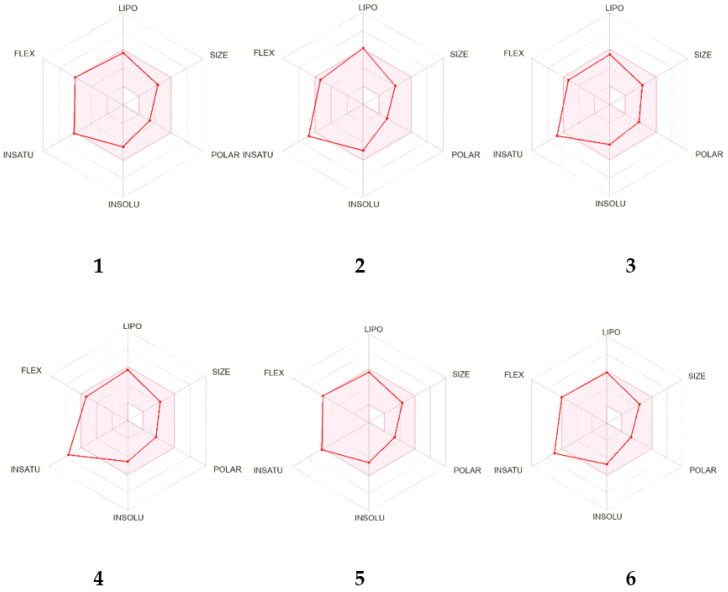
In silico study for drug-likeness using the SwissADME tool on Compounds **1**–**6** (red area represents the optimal range for each property).

To evaluate the potential of Compounds **1**–**6** as suitable compounds for the conduction of anti-inflammatory in vivo assays, an in silico study using the SwissADME tool was performed [14]. This approach investigates several parameters, such as physicochemistry, PK, and drug-likeness. As can be observed in the bioavailability radar (Figure 2), the identified compounds in HEB demonstrated good adherence to all evaluated parameters.

ADME parameters can be determined by in silico studies based on calculated physicochemical standards and provide useful information on lipophilicity, bioavailability, drug-likeness parameters, and different physicochemical properties, such as water solubility, molecule size, polarity, and flexibility, as also indicated in Table 1.

The log *p* values of Compounds **1**–**6** ranged from 3.69 to 4.75 and were therefore in accordance with Lipinski (log P_o/w_ ≤ 5), Ghose (log P_o/w_ ≤ 5.6), and Egan (log P_o/w_ ≤ 5.8) rules. The results regarding the number of hydrogen acceptors and donors correspond to Lipinski’s rule, suggesting that all compounds exhibited oral bioavailability. However, Veber presents two essential parameters for molecules to be administered orally, which are a topological polar surface area (TPSA) ≤ 140 Å^2^ and the number of rotatable connections. Compounds **2**–**6** showed TPSAs ranging from 47.92 to 68.15 Å2, while Compound **1** had a TPSA of 36.92 Å^2^, suggesting strong prospects for oral use. Water solubility is an important feature for the absorption and distribution of a drug in an organism. The log S values (determined using the Ali method) [15] calculated for Compounds **3**–**6** were inferior that 6, indicating that they had moderate solubility; however, for Compounds **1** and **2**, these values were at the limit, calculated as −6.04 and −5.97, respectively. Finally, no alert for PAINS was observed for the structures of Compounds **1**–**6**. Thus, the results of in silico analysis supported the conduction of assays for the evaluation of anti-inflammatory activity in crude hexane extract from *E. paniculata*, composed of Compounds **1**–**6** and its main compound, methyldehydrodieugenol B (**1**), due to their good absorption and biodistribution in vivo.

### 2.3. Evaluation of Inflammatory Response Induced by HEB and Methyldehydrodieugenol B in Sponge Implants

**Figure 3:**
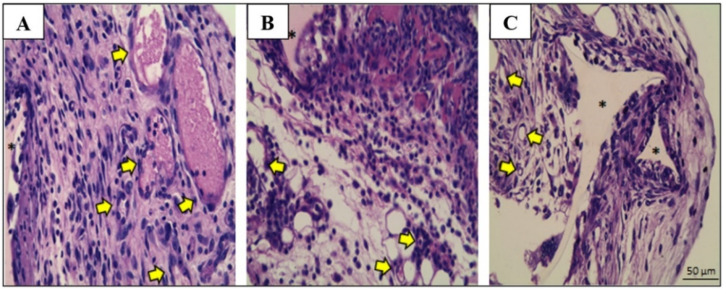
Histological analysis of 9-day-old implants in hematoxylin-and-eosin-stained sections, at 400× magnification (scale bar: 50 µm): representative sections of control (**A**), HEB-treated implants (**B**), and methyldehydrodieugenol-B-treated implants (**C**). The newly formed granulation tissue is composed of a mixture of inflammatory cells, fibroblasts, and numerous blood vessels (yellow arrows). Asterisks (*) indicate a pore of the synthetic matrix (triangular shape).

Subcutaneous sponge implants are responsible for inducing the development of fibrovascular tissue, marked by an intense infiltration of inflammatory cells, caused by the formation of new blood vessels (angiogenesis) and the migration and proliferation of fibroblasts with the synthesis of extracellular matrix components. A reduction in cellular infiltration and the number of blood vessels occurred in implants treated with methyldehydrodieugenol B alone when compared to the control group. In contrast, in the implants treated with HEB, it was possible to observe an accumulation of inflammatory cells (Figure 3).

**Figure 4:**
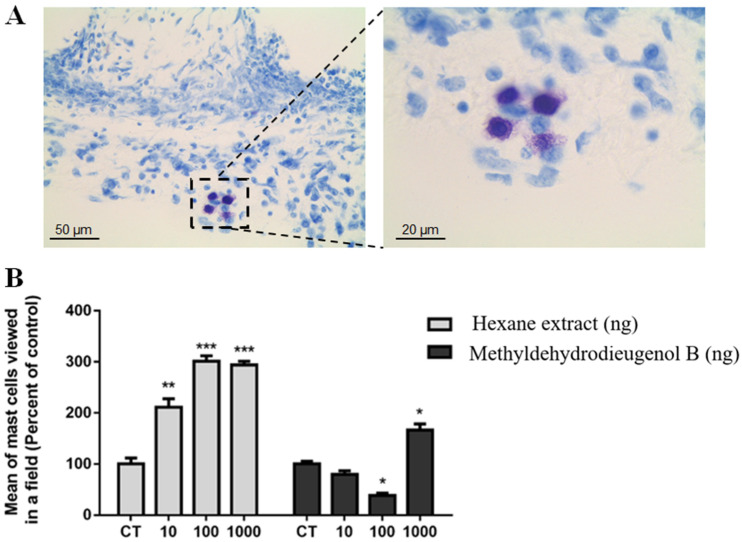
Histological analysis of the number of mast cells in 9-day-old implants. (**A**) Photomicrographs of toluidine-blue-stained sections (5 μm) at 400× magnification (scale bar: 50 μm) showing histological analyses of the number of mast cells after intraimplant injections in the control group. The squared region is depicted at a higher level of magnification (×1000, scale bar: 20 μm) in the following image, showing the mast cells. (**B**) Graphical representation of mean of mast cells of control, hexane extract and methyldehydrodieugenol B. * *p* < 0.05, ** *p* < 0.01, and *** *p* < 0.001 vs. CT.

The inflammatory response associated with the presence of spongy matrices was measured indirectly by the activities of MPO and NAG enzymes and by the quantification of mast cells present in fibrovascular tissue. Mast cells (Figure 4A) are cells in the final stages of differentiation, derived from hematopoietic progenitors, present in different systems of the body. They play an important role in regulating the inflammatory response and in later repair, being a source of bioactive mediators, such as histamine and prostaglandins; potent vasodilators; and cytokines and chemokines associated with the recruitment of cells from the bloodstream, leading to the accumulation of leukocytes near the inflammatory site [16,17,18]. The results following the intraimplant administration of HEB elucidated this relationship. Following treatment with HEB, for all tested doses, there was a very high increase in the average number of mast cells, reaching a 201.24% increase after the 100 ng dose when compared to the control group (CT).

Treatment with methyldehydrodieugenol B showed a reduction in the number of mast cells at the 100 ng dose (61.91%) but a considerable increase of 63% at a dose of 1000 ng when compared to the CT group (Figure 4B). The reduction in the number of mast cells after treatment with methyldehydrodieugenol B (**1**) at a dose of 100 ng may have contributed to the lower accumulation of inflammatory cells close to the fibrovascular tissue formed. In addition, the reduction in the number of mast cells in the treated group supports the potential therapeutic use of methyldehydrodieugenol B in the treatment of allergies [19,20] and hyperlipidemia [21], diseases related to a very large increase in the number of mast cells. The effects of the administration of other neolignans on mast cell degranulation and activation has been investigated by other groups [20]. Licarin A, for example, was able to control the secretion of pro-inflammatory mediators and the release of histamine by these cells. The pharmacological mechanisms involve changes in intracellular Ca^2+^ influx and the activation of signaling pathways (e.g., PKCα/βII and p38 MAPK) responsible for controlling such events [22]. Despite the increase in the number of mast cells observed in implants treated with 1000 ng of methyldehydrodieugenol B, this increase was not enough to cause an increase in neutrophil or macrophage infiltration, as observed in the evaluation of MPO and NAG enzyme activity, respectively.

**Figure 5:**
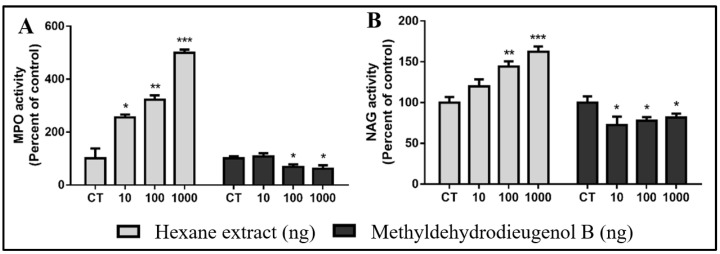
HEB and methyldehydrodieugenol B treatment in all tested concentrations downregulate inflammatory markers. (**A**) Myeloperoxidase activity (MPO) and (**B**) *N*-acetyl-β-D-glucosaminidase activity (NAG). * *p* < 0.05, ** *p* < 0.01, and *** *p* < 0.001 vs. CT.

The neutrophil contents of the implants were measured indirectly by assessing the activity of the myeloperoxidase enzyme (MPO), which is present in large quantities inside the azurophilic granules of these cells. Treatment with methyldehydrodieugenol B significantly reduced MPO activity at doses of 100 and 1000 ng when compared to the CT group (31.73 and 38.41%, respectively). However, when the animals were treated with HEB, at all doses tested, there was a very high increase in MPO activity when compared to the CT group (155.85, 235.40, and 358.78% for 10, 100, and 1000 ng, respectively; Figure 5A). The compound methyldehydrodieugenol B attenuates the activity of neutrophils, as previously demonstrated for dehydrodieugenol B [6]. Our results corroborate those studies that demonstrated similar effects of treatment with isolated phenylpropanoids in in vivo models [7,8,23]. For instance, the oral administration of anethole reduced inflammatory edema, neutrophil accumulation, and MPO activity in different models of acute inflammation [7]. Macrophage content was evaluated based on the level of NAG enzyme activity. Animals treated with HEB showed an increase in NAG activity following the 100 and 1000 ng doses when compared to the CT group (144.48 and 162.49%, respectively). However, when animals were treated with methyldehydrodieugenol B, a significant reduction in NAG activity was observed after treatment with all doses when compared to the CT group (27.58, 22.46, and 18.34%; Figure 5B).

### 2.4. Antiangiogenic Activity of HEB and Methyldehydrodieugenol B

**Figure 6:**
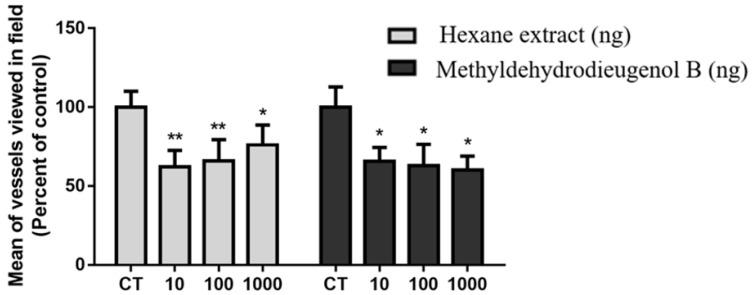
Effects of treatments with hexane extract and methyldehydrodieugenol B on angiogenic markers (mean of number of vessels). The values shown are the means (±SEM). * *p* < 0.05 and ** *p* < 0.01 vs. CT.

In this work, the effects of HEB and methyldehydrodieugenol B on angiogenesis were also evaluated according to biochemical and morphological parameters. Angiogenesis was assessed directly by blood vessel count in the histological sections. The numbers of blood vessels formed in implants treated with HEB and methyldehydrodieugenol B were lower when compared to the CT group (37.89, 34.02, and 23.91% for HEB and 34.29, 37.15, and 40.00% for methyldehydrodieugenol B at 10, 100, and 1000 ng, respectively; Figure 6). Both HEB and the compound methyldehydrodieugenol B had an antiangiogenic effect on sponge-induced fibrovascular tissue when compared to the control group.

Excessive or insufficient angiogenesis is associated with major classes of chronic disease. Excessive angiogenesis is associated with cancer, psoriasis, age-related macular degeneration, and arthritis [24,25,26]. Thus, drugs with antiangiogenic effects, as observed with methyldehydrodieugenol B, are considered a potential target for antitumor therapy [27].

### 2.5. Anti-Fibrogenic Effects of HEB and Methyldehydrodieugenol B

**Figure 7:**
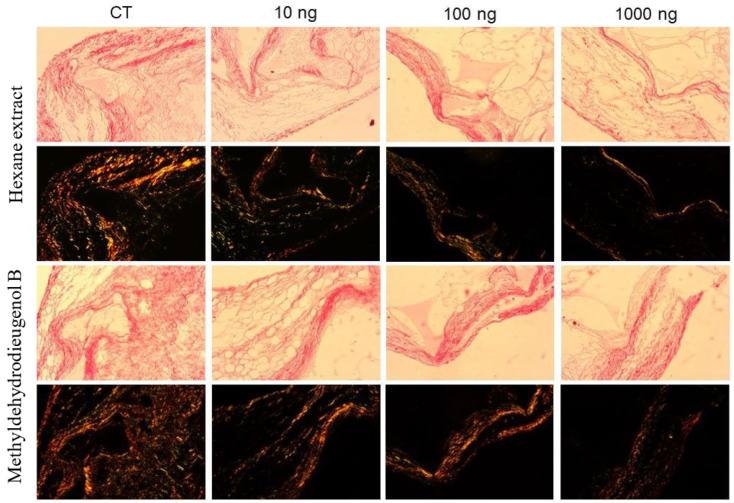
Histological analysis of collagen deposition after treatment with HEB and methyldehydrodieugenol B. Representative histological sections were stained with picrosirius red, with and without the polarized light filter. Under this filter, type III collagen fibers can be distinguished from type I fibers (red/orange).

To evaluate the effects of treatments with HEB and methyldehydrodieugenol B on soluble collagen content, the total collagen and type I and III collagen fibers were evaluated. Collagen synthesis and deposition in implant-induced fibrovascular tissue were assessed using the biochemical dosage of soluble collagen and the histological staining technique with picrosirius red (Figure 7).

**Figure 8:**
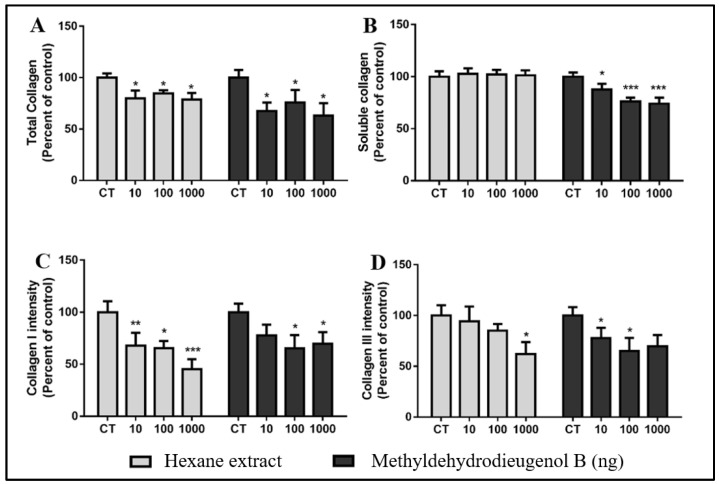
Histological analysis of collagen deposition after treatment with HEB and methyldehydrodieugenol B. Graphical representations of (**A**) total collagen density, (**B**) type I collagen density, (**C**) type III collagen density, and (**D**) soluble collagen. * *p* < 0.05, ** *p* < 0.01, and *** *p* < 0.001 vs. CT.

In implants treated with HEB and the compound methyldehydrodieugenol B, at all doses, there was a reduction in the deposition of total collagen (Figure 8A) when compared to the CT group (20.31, 15.25, and 21.31% for HEB and 32.59, 24.04, and 37.04% for methyldehydrodieugenol B at 10, 100, and 1000 ng, respectively). The same behavior was observed in the collagen intensities of types I and III (Figure 8C,D). These results show that treatments with HEB and methyldehydrodieugenol B cause an antifibrogenic effect (a decrease in the amount of collagen). In implants treated with HEB, there were no significant differences in the content of soluble collagen when compared to the CT group at any dose. However, in implants treated with methyldehydrodieugenol B, there were reductions in collagen for all doses tested when compared to CT (12.22, 23.90, and 25.91% for 10, 100, and 1000 ng, respectively; Figure 8B).

Previous studies testing phenylpropanoids or extracts enriched with these compounds have also demonstrated antifibrogenic effects, such as those observed in the results of HEB and methyldehydrodieugenol B treatment [28,29,30,31]. Despite the need for the deposition of new extracellular matrix constituents after an injury, which leads to the restoration of tissue architecture and homeostasis, a prolonged or severe injury can exacerbate this process, leading to the appearance of fibrosis. This accumulation of matrix constituents, especially collagen, can lead to the dysfunction or failure of a particular organ. Cellular components and mediators of the inflammatory response play an important role in the development of fibrosis; therefore, targeting the main inflammatory pathways can also be beneficial in the treatment of this condition [32,33,34].

## 3. Materials and Methods

### 3.1. General Experimental Procedures

One-dimensional (^1^H, ^13^C and DEPT) and two-dimensional (COSY and HSQC) NMR spectra were recorded on a Bruker Ascend™ 400 Avance III HD spectrometer (9.4 Tesla), operating at 400 MHz (^1^H) and 100 MHz (^13^C), at 30 °C. CDCl_3_ and TMS (Sigma-Aldrich) were used as the solvent as the internal standard, respectively. Column chromatography (CC) was performed with silica gel (230–400 mesh, Merck) while thin layer chromatography (TLC) separations were carried out on silica gel 60 PF254 (Merck).

### 3.2. Plant Material

Branches of *E. paniculata* were collected in November 2014, following the procedures described by Souza et al. [6] (voucher specimens EM 335 and EM 355) and registered under the code AD99BA0 in SisGEN in Brazil.

### 3.3. Extraction of Plant Material

Fresh branches from *E. paniculata* were dried at 35 °C and powdered to afford 11.6 g of plant material, which was extracted with hexane (8 × 100 mL) at room temperature. Combined extracts were concentrated under reduced pressure (40 °C) to yield 909 mg hexane extract (HEB).

### 3.4. Chemical Analysis of Hexane Extract from Branches (HEB) of E. paniculata

HEB was submitted to HPLC-ESI/MS using a Shimadzu CBM-20A HPLC system coupled to a Mass Spectrometer MAXIS 3G (Bruker Daltonics) with an electrospray ionization source (ESI). HPLC separation procedures were conducted using the following parameters: Supelco Ascentis C_18_ 5 µm column (250.0 mm × 4.6 mm × 5 µm), mobile phase composed by Milli-Q H_2_O (A) and MeOH (B); gradient: 50% B (0 min), 100% B (0–35 min), 100% B (35–40 min), 50% B (40–41 min), and 50% B (41–45 min). The ionization conditions included a nebulizer pressure of 2 Bar, an injection flow rate of 8 L/min, secant gas maintained at 250°C, and an energy of 4.5 kV in the capillary.

In order to isolate the main metabolite in HEB, part of the crude extract (400 mg) was subjected to silica gel column chromatography, eluted with increasing amounts of EtOAc in hexane (9:1, 7:1, 5:1, 3:1, and 1:1) and pure EtOAc to give 162 fractions (10 mL each) which were pooled together in 11 groups (A–K) after TLC analysis. Part of Group C (56 mg) was purified by silica gel column chromatography eluted with increasing amounts of EtOAc in CH_2_Cl_2_ (20:1, 14:1, 10:1, 6:1, 3:1, and 1:1) to afford methyldehydrodieugenol B (12 mg) as a yellowish oil.

^1^H NMR (CDCl_3_) δ 6.83 (d, *J* = 7.1 Hz, H-5′), 6.82 (d, *J* = 2.1 Hz, H-2′), 6.70 (dd, *J* = 7.8 and 2.0 Hz, H-6′), 6.29 (d, *J* = 2.0 Hz, H-2), 6.45 (d, *J* = 2.0 Hz, H-6), 5.90 (m, H-8 and H-8′), 5.06 (m, H-9 and H-9′), 3.88 (s, H-11 and H-10′), 3.84 (s, H-10), 3.37 (d, *J* = 6.4 Hz, H-7′), and 3.24 (d, *J* = 6.1 Hz, H-7); ^13^C NMR (CDCl_3_) δ 153.7 (C, C-3′), 150.8 (C, C-4′), 150.8 (C, C-5), 144.3 (C, C-3), 138.2 (C, C-4), 137.6 (CH, C-8), 137.3 (CH, C-8′), 136.2 (C, C-1′), 135.7 (C, C-1), 120.9 (CH, C-6′), 119.6 (C, C-5′), 116.1 (CH_2_, C-9/C-9′), 113.2 (CH, C-2′), 111.5 (CH, C-6), 107.5 (CH, C-2), 61.2 (CH_3_, C-11), 56.3 (CH_3_, C-10), 56.2 (CH_3_, C-10′), 40.3 (CH_2_, C-7′), 40.2 (CH_2_, C-7). ESI-HRMS *m/z* 363.1585 [M + Na]^+^ (calculated for C_21_H_24_O_4_Na 363.1572).

### 3.5. In Silico Analysis of Pharmacokinetic Parameters

The physicochemical properties, lipophilicity, water solubility, and drug-likeness parameters of methyldehydrodieugenol B were obtained using the free online software SwissADME (www.swissadme.ch, accessed on 17 December 2022) (Swiss Institute of Bioinformatics^®^, Lausanne, Switzerland) [14], developed and maintained by the Molecular Modeling Group of the Swiss Institute of Bioinformatics. On this website, 2D structural models of analyzed compounds were drawn in the molecular sketcher into ChemAxon’s Marvin JS window and transferred into a SMILES (simplified molecular-input line-entry system) format to predict suitable properties. These results were analyzed using the rules of Lipinski [35], Ghose [36], Veber [37], and Egan [38], which are used to evaluate the pharmacokinetic characteristics of drug candidates.

### 3.6. Ethics Statement

All animal procedures were approved by the Ethics Commission on Animal Use (CEUA) from the Federal University of Uberlandia (CEUA 083/2017).

### 3.7. Animals

Male C57BL/6 mice (128 animals) were kept in idolators in groups of five, until it was time for the surgical implantation of the sponges. After surgery, the animals were individualized. Standard temperature (22 °C) and relative humidity (60–65%) conditions and a 12 h light/dark cycle were maintained throughout the experiment. The animals also had free access to water and food.

### 3.8. Surgical Implantation of the Sponge Discs and Treatment Protocol

As previously described [39,40], polyether-polyurethane sponges (8 mm diameter × 4 mm thick; Vitafoam Ltd., Manchester, UK) were aseptically implanted into the dorsum of the mice, near the interscapular region, through a dorsal midline incision (1 cm long). In order to sterilize the material to be implanted, the sponge discs were kept overnight in EtOH 70% (*v*/*v*) and boiled in distilled H_2_O (for 30 min) moments before surgery. With the animals already anesthetized (by a mixture of 100 mg/kg of ketamine and 10 mg/kg of xylazine), we performed trichotomy and asepsis of the dorsal region with EtOH (70% *v*/*v*). A single sponge disc was inserted into each animal. During a period of nine consecutive days, starting immediately after the sponge disc implantation surgery, the animals received intraimplant injections containing 10 μL of PBS solution containing 0.5% DMSO (*v*/*v*) (control group–CT) or 0.01, 0.1, or 1 mg of the HEB or methyldehydrodieugenol B per day, with both concentrations diluted in 10 μL of PBS solution containing 0.5% DMSO and injected in the central region of the implants (intraimplant) using an insulin syringe (BD Eclipse). On the 9th day post implantation, the animals were euthanized, and the implants were removed and processed for biochemical and histological analyses.

### 3.9. Myeloperoxidase (MPO) and N-acetyl-β-D-glucosaminidase (NAG) Activities

Myeloperoxidase (MPO) activity was used to measure the number of neutrophils in the implants [41,42]. Initially, the implants were weighed, homogenized (0.015 M Na-EDTA, 0.02 M Na_3_PO_4_, 0.1 M NaCl, pH 4.7 buffer, Sigma-Aldrich, Saint Louis, United States), and centrifuged (10 min, 12,000× *g*, 4° C). Then, the pellets were resuspended (pH 5.4, 0.05 M Na_3_PO_4_ buffer, 0.5% hexadecyltrimethylammonium bromide (HTAB), Sigma-Aldrich) and subjected to three freeze–thaw cycles using liquid nitrogen. 3,3′-5,5′-tetramethylbenzidine (TMB) was prepared in dimethylsulfoxide (DMSO) and H_2_O_2_ (0.3 mM) in sodium phosphate buffer (pH 6.0; final concentration of 1.6 mM). This solution was used to evaluate the levels of MPO activity in the supernatant samples. To end the reaction, 50 μL H_2_SO_4_ (4 M) was added, and the variation in absorbance (450 nm) was measured. The results were expressed as variations in the optical density/g of wet tissue (OD/g). The levels of *N*-acetyl-β-D-glucosaminidase (NAG) were measured to quantify the infiltration of mononuclear cells [41,42]. A NaCl solution (0.9% *w*/*v*) containing 0.1% *v*/*v* of Triton X-100 (Promega, Madison, United States) was used to homogenize the implants, which were then centrifuged for 10 min (3000× *g*; at 4 °C). A 100 µL sample of the resulting supernatant was added to a solution of 100 μL of *p*-nitrophenyl-*N*-acetyl-β-D-glucosaminide (Sigma-Aldrich, Saint Louis, United States) prepared in phosphate/citrate buffer (pH 4.5, 0.1 M Na_2_HPO_4_, 0.1 M citric acid) for 30 min (final concentration of 2.24 mM). To end the reaction, 100 μL 0.2 M glycine buffer (pH 10.6) was added. The absorption of the solution was measured at 400 nm, and the results of substrate hydrolysis were expressed in nmol/mg of wet tissue.

### 3.10. Histological Analysis and Staining

The implants (n = 6 for each group, Table S3) were excised carefully, fixed in methacarn (60% MeOH, 30% CHCl_3_, 10% acetic acid, *v*/*v*) at 4 °C for 24 h, and embedded in paraffin. The analyses were performed as described in previous studies [42,43]. Briefly, twenty-five sequential fields were captured from the histological sections (5 µm) and stained with hematoxylin and eosin (H and E) or toluidine blue using a LEICA ICC50 microcamera. From these images, the presence of inflammatory infiltrate and the mean numbers of blood vessels and mast cells near the sponge implants were evaluated. Histological sections stained with picrosirius red were analyzed without and with the presence of a polarized light filter, and images were captured using an Optcam microcamera system to produce images to be analyzed with a Nikon TS 100 microscope (15 sequential fields). The images obtained with the polarization filter allow for the distinction between type III collagen (usually corresponding to thinner collagen fibers) and type I collagen (thicker fibers), displayed in green and red/orange, respectively. Images were evaluated using Image J.

### 3.11. Statistical Analysis

All data were expressed as mean ± SEM. Comparisons between groups were made using ANOVA followed by Newman–Keuls correction factor as a posttest. The GraphPad Prism program, version 6.0, was used to perform the statistical analysis and graph construction. For *p* values < 0.05, the differences between means were considered significant.

## 4. Conclusions

The hexane extract of branches (HEB) from *E. paniculata* was analyzed for the first time concerning chemical composition, as well as its effect on the components of the inflammatory response induced by sponge implants in vivo. HPLC-ESI/MS analysis allowed for the identification of six chemically related neolignans, with methyldehydrodieugenol B being the main compound found in the extract. The in silico analysis of different pharmacokinetic parameters suggested good solubility, sound absorption, and biodistribution for Compounds **1**–**6** in vivo. The anti-inflammatory activity of both HEB and purified methyldehydrodieugenol B (**1**) were evaluated. The results obtained demonstrated that the treatment of mice with HEB caused inflammatory, anti-fibrogenic, and anti-angiogenic effects. In contrast, treatment with methyldehydrodieugenol B caused anti-inflammatory, anti-fibrogenic, and anti-angiogenic effects. The obtained results demonstrate the therapeutic potential of HEB but especially methyldehydrodieugenol B in the treatment of pathologies associated with inflammation and angiogenesis, including chronic wounds.

## Figures and Tables

**Table 1:** In silico physicochemical properties and ADMET predictions for Compounds **1**–**6**.

Parameters	Compounds					
	1	2	3	4	5	6
Physicochemical Properties						
Num. Heavy atoms	25	24	25	25	26	26
Fraction Csp^3^	0.24	0.20	0.20	0.15	0.24	0.19
Num. Rotatable Bonds	9	8	8	8	9	9
Num. H-bonds acceptors	4	4	5	5	5	5
Num. H-bonds donors	0	1	2	1	1	0
Molar Refractivity	100.65	96.18	97.34	96.60	101.81	101.07
TPSA ^a^/Å^2^	36.92	47.92	68.15	64.99	57.15	53.99
Lipophilicity						
log P_o/w_ ^b^	4.75	4.44	3.69	3.84	4.06	4.20
Water Solubility						
log S	−6.04	−5.97	−5.21	−5.46	−5.31	−5.57
Class ^c^	PS	MS	MS	MS	MS	MS
Drug-likeness						
Lipinski ^d^	Yes	Yes	Yes	Yes	Yes	Yes
Ghose ^e^	Yes	Yes	Yes	Yes	Yes	Yes
Veber ^f^	Yes	Yes	Yes	Yes	Yes	Yes
Egan ^g^	Yes	Yes	Yes	Yes	Yes	Yes
Bioavailability Score	0.55	0.55	0.55	0.55	0.55	0.55
Medicinal Chemistry						
PAINS	0 alert	0 alert	0 alert	0 alert	0 alert	0 alert

^a^ TPSA: topological polar surface area; ^b^ log P_o/w_ = partition coefficient between *n*-octanol and water; ^c^ Class: insoluble (I) < −10 < poor soluble (PS) < −6 < moderately soluble (MS) < −4 < soluble (S) < −2 < very soluble (VS) < 0 < highly soluble (HS); ^d^ Lipinski = MM ≤ 500; log P_o/w_ ≤ 5; H-bond donors ≤ 5; H-bond acceptors ≤ 10; ^e^ Ghose = 180 ≤ MM ≤ 480; 20 ≤ No. of atoms ≤ 70; 40 ≤ Molar Refractivity ≤ 130; −0.4 ≤ log P_o/w_ ≤ 5.6; ^f^ Veber = Num. Rotatable Bonds ≤ 10; TPSA ≤ 140 Å^2^; ^g^ Egan = log P_o/w_ ≤ 5.88; TPSA ≤ 131.6 Å^2^.

## Data Availability

Not applicable.

## Supplementary Materials

The following supporting information can be downloaded at: https://www.mdpi.com/article/10.3390/molecules28135247/s1, Figures S1–S8: NMR spectra of the compound methyldehydrodieugenol B, Figure S9: HPLC analysis of HEB, Figures S10–S21: MS spectra of Compounds **1**–**6**.
