# Supplementary material for: An In Vivo Assessment of the Effect of Hexane Extract from Endlicheria paniculata Branches and Its Main Compound, Methyldehydrodieugenol B, on Murine Sponge-Induced Inflammation
Source: Molecules. 2023 Jul 6;28(13):5247. doi: 10.3390/molecules28135247 (PMC10343220; doi:10.3390/molecules28135247)
Supplement: Supplementary file 1 [file molecules-28-05247-s001.zip › molecules-2435887-supplementary.pdf]

Supplementary Material

An *In Vivo* Assessment of the Effect of Hexane Extract from *Endlicheria paniculata* Branches and Its Main Compound, Methyldehydrodieugenol B, on Murine Sponge-Induced Inflammation

Bruno Antonio Ferreira [1,2,†], Rafael Aparecido Carvalho Souza [3,†], Francyelle Borges Rosa de Moura [4,5], Tiara da Costa Silva [3], Tais da Silva Adriano [3], Eduardo de Faria Franca [3], Raquel Maria Ferreira de Sousa [3], Fernanda de Assis Araújo [1], João Henrique Ghilardi Lago [2,*] and Alberto de Oliveira [3,*]

[1] Department of Physiological Sciences, Federal University of Uberlandia, Uberlandia, 38408-100, Brazil; bruno.antonioferreira70@gmail.com (B.A.F.); folaraujo@gmail.com (F.d.A.A.)
[2] Center for Natural and Human Sciences, Federal University of ABC, Santo Andre, 09210-180, Brazil
[3] Institute of Chemistry, Federal University of Uberlandia, Uberlandia, 38408-100, Brazil; rafasouza27@ufu.br (R.A.C.S.); tiaracosta@ufu.br (T.d.C.S.); taisadriano9@gmail.com (T.d.S.A.); eduardofranca@ufu.br (E.d.F.F.); rsousa@ufu.br (R.M.F.d.S.)
[4] Department of Cell Biology, Histology and Embryology, Federal University of Uberlandia, Uberlandia, 38408-100, Brazil; francyelle.moura@ufcat.edu.br
[5] Department of Biological Sciences, Federal University of Catalao, Catalao, 75704-020, Brazil
* Correspondence: joao.lago@ufabc.edu.br (J.H.G.L.); alberto@ufu.br (A.d.O.)
† These authors contributed equally to this work.

Tables

Table S1. Identification of Compounds **1–6** in HEB of *E. paniculata* by HPLC-ESI-MS. 3
Table S2. ^{1}H NMR (400 MHz), ^{13}C (100 MHz), COSY and HSQC (in $CDCl_3$) data of **1**. 3
Table S3. Distribution, description and number of mice per analysis. 4

Figures

Figure S1. ^{1}H NMR spectrum (400 MHz, $CDCl_3$) of **1**. 4
Figure S2. ^{13}C NMR spectrum (100 MHz, $CDCl_3$) of **1**. 5
Figure S3. DEPT135 NMR spectrum from enlarged 40 to 155 ppm (δ, $CDCl_3$, 100 MHz) of **1**. 5
Figure S4. COSY contour map (δ, $CDCl_3$, 100 MHz) of **1**. 6
Figure S5. HSQC contour map (δ, $CDCl_3$, 400 and 100 MHz) of **1**. 6
Figure S6. ^{1}H NMR spectrum (region from 3 to 4 ppm) (400 MHz, $CDCl_3$) of **1**. 7
Figure S7. ^{1}H NMR spectrum (region from 5 to 6 ppm) (400 MHz, $CDCl_3$) of **1**. 7
Figure S8. ^{1}H NMR spectrum (region from 6 to 7 ppm) (400 MHz, $CDCl_3$) of **1**. 8
Figure S9. HPLC analysis of hexane extract from branches (HEB) of *E. paniculata*. 8
Figure S10. HPLC–ESI–MS spectrum (positive mode) of **1** at 27.0 min. 9
Figure S11. HPLC–ESI–MS/MS spectrum (positive mode) of **1** at 27.0 min. 9
Figure S12. HPLC–ESI–MS spectrum (positive mode) of Compound **2** at 24.1 min. 9
Figure S13. HPLC–ESI–MS/MS spectrum (positive mode) of Compound **2** at 24.1 min. 10
Figure S14. HPLC–ESI–MS spectrum (positive mode) of Compound **3** at 15.1 min. 10
Figure S15. HPLC–ESI–MS/MS spectrum (positive mode) of Compound **3** at 15.1 min. 10
Figure S16. HPLC–ESI–MS spectrum (positive mode) of Compound **4** at 16.9 min. 11
Figure S17. HPLC–ESI–MS/MS spectrum (positive mode) of Compound **4** at 16.9 min. 11
Figure S18. HPLC–ESI–MS spectrum (positive mode) of Compound **5** at 18.1 min. 11
Figure S19. HPLC–ESI–MS/MS spectrum (positive mode) of Compound **5** at 8.1 min. 12
Figure S20. HPLC–ESI–MS spectrum (positive mode) of Compound **6** at 21.3 min. 12
Figure S21. HPLC–ESI–MS/MS spectrum (positive mode) of Compound **6** at 21.3 min. 12

Table S1. Identification of Compounds **1–6** in HEB of *E. paniculata* by HPLC-ESI-MS.

R_t / min	*m*/*z* [M + Na]$^+$	Error (ppm)	Molecular formula	Identified compound
15.1	365.1369	2.7	$C_{20}H_{22}O_5$	1-[(7*R*)-hydroxy-8-propenyl]-3-[3′-methoxy-1′-(8′-propenyl)phenoxy]-4-hydroxy-5-methoxybenzene (**3**)
16.9	363.1213	2.7	$C_{20}H_{20}O_5$	1-(7-oxo-8-propenyl)-3-[3′-methoxy-1′-(8′-propenyl)phenoxy]-4-hydroxy-5-methoxybenzene (**4**)
18.1	379.1533	4.5	$C_{21}H_{24}O_5$	1-[(7*R*)-hydroxy-8-propenyl]-3-[3′-methoxy-1′-(8′-propenyl)phenoxy]-4,5-methoxybenzene (**5**)
21.3	377.1372	3.4	$C_{21}H_{22}O_5$	1-(7-oxo-8-propenyl)-3-[3′-methoxy-1′-(8′-propenyl)phenoxy]-4,5-dimethoxybenzene (**6**)
24.1	349.1424	4.0	$C_{20}H_{22}O_4$	Dehydrodieugenol B (**2**)
27.0	363.1585	4.0	$C_{21}H_{24}O_4$	Methyldehydrodieugenol B (**1**)

Table S2. ^{1}H NMR (400 MHz), ^{13}C (100 MHz), COSY and HSQC (in $CDCl_3$) data of **1**.

Position	^{1}H (mult[1], *J* in *Hz*)	^{13}C	COSY	HSQC
**1**	-	135.7	-	-
**2**	6.28 (d, 2.0)	107.5	-	C-2
**3**	-	144.3	-	-
**4**	-	138.2	-	-
**5**	-	150.8	-	-
**6**	6.49 (d, 2.0)	111.5	-	C-6
**7**	3.37 (d, 6.4)	40.2	H-8	C-7
**8**	5.90 (m)	137.6	H-7, H-9	C-8
**9**	5.06 (m)	116.1	H-8	C-9
**10**	3.84 (s)	56.3	-	C-10
**11**	3.88 (s)	61.2	-	C-11
**1′**	-	136.2	-	-
**2′**	6.81 (d, 2.1)	113.2	-	C-2′
**3′**	-	153.7	-	-
**4′**	-	150.8	-	-
**5′**	6.83 (d, 7.1)	119.6	H-6′	C-5′
**6′**	6.70 (dd, 7.8 and 2.0)	120.9	H-5′	C-6′
**7′**	3.24 (d, 6.1)	40.3	H-8′	C-7′
**8′**	5.90 (m)	137.3	H-7′, H-9′	C-8′
**9′**	5.06 (m)	116.1	H-8′	C-9′
**10′**	3.88 (s)	56.2	-	C-10′

Note: [1]multiplicity of signals: s = singlet; d = doublet; dd = double-doublet; m = multiplet.

Table S3. Distribution, description and number of mice per analysis.

Groups	Descriptions	Number of mice per analysis	
		**Biochemical analysis**	**Histological analysis**
CO	The animals received intraimplant treatment, 10 μL of 0.5% DMSO (vehicle), for 9 consecutive days	*n* = 10	*n* = 6
0.1 μg	Animals treated with 10 ng of HEB, diluted in 10 μL 0.5% DMSO (intraimplant injections for 9 consecutive days)	*n* = 10	*n* = 6
1 μg	Animals treated with 100 ng of HEB, diluted in 10 μL 0.5% DMSO (intraimplant injections for 9 consecutive days)	*n* = 10	*n* = 6

10 μg	Animals treated with 1000 ng of HEB, diluted in 10 μL 0.5% DMSO (intraimplant injections for 9 consecutive days)	$n = 10$	$n = 6$
			**Total = 64**

Groups	Descriptions	Biochemical analysis	Histological analysis
CO	The animals received intra-implant treatment, 10 μL 0.5% DMSO (vehicle), for 9 consecutive days	$n = 10$	$n = 6$
0.1 μg	Animals treated with 10 ng of methyldehydrodieugenol B, diluted in 10 μL 0.5% DMSO (intraimplant injections for 9 consecutive days)	$n = 10$	$n = 6$
1 μg	Animals treated with 100 ng of methyldehydrodieugenol B, diluted in 10 μL 0.5% DMSO (intraimplant injections for 9 consecutive days)	$n = 10$	$n = 6$
10 μg	Animals treated with 1000 ng of methyldehydrodieugenol B, diluted in 10 μL 0.5% DMSO (intraimplant injections for 9 consecutive days)	$n = 10$	$n = 6$
			**Total = 64**

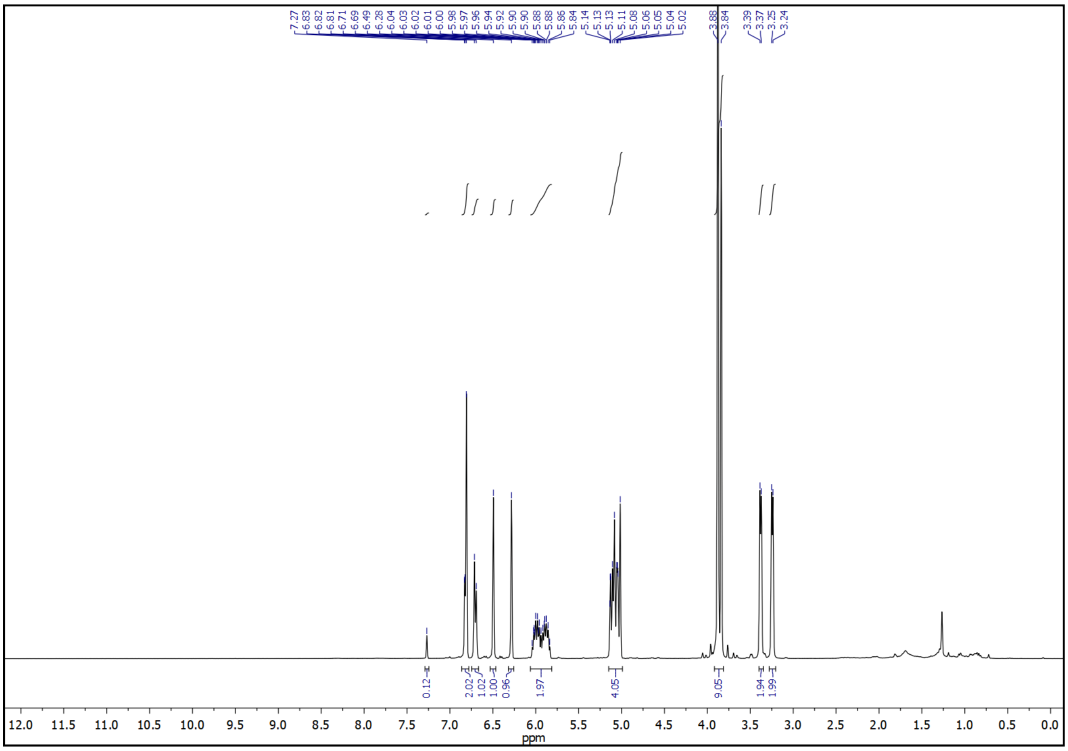

Figure S1. ^{1}H NMR spectrum (400 MHz, $CDCl_3$) of **1**.

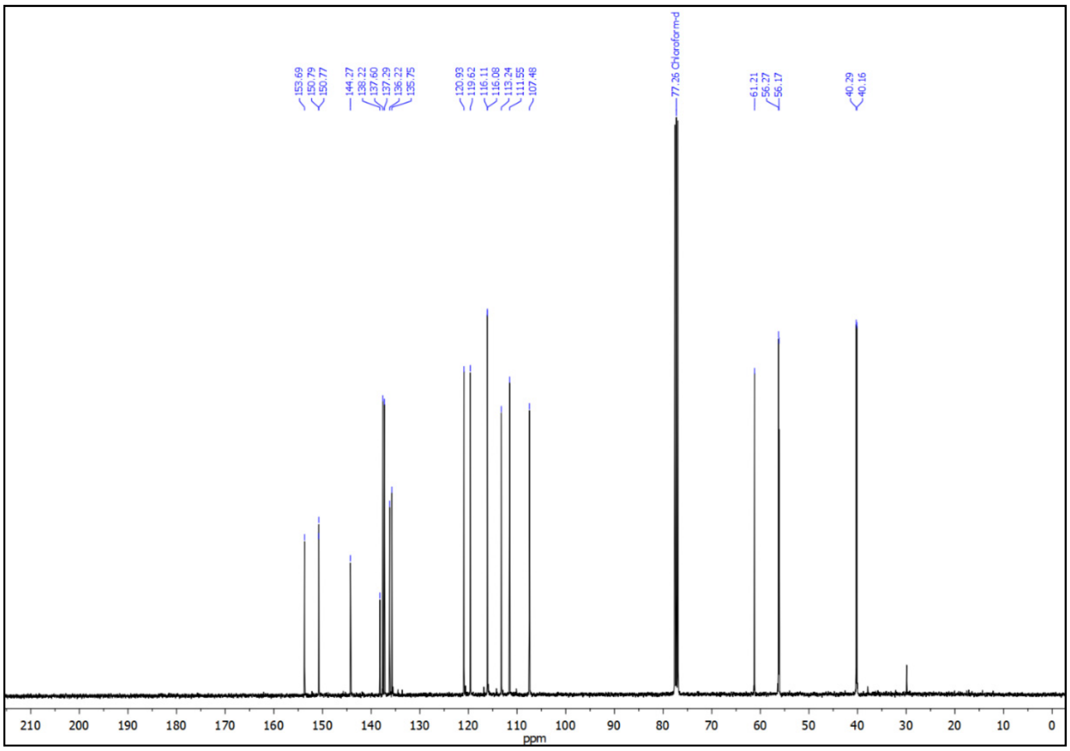

Figure S2. ^{13}C NMR spectrum (100 MHz, $CDCl_3$) of **1**.

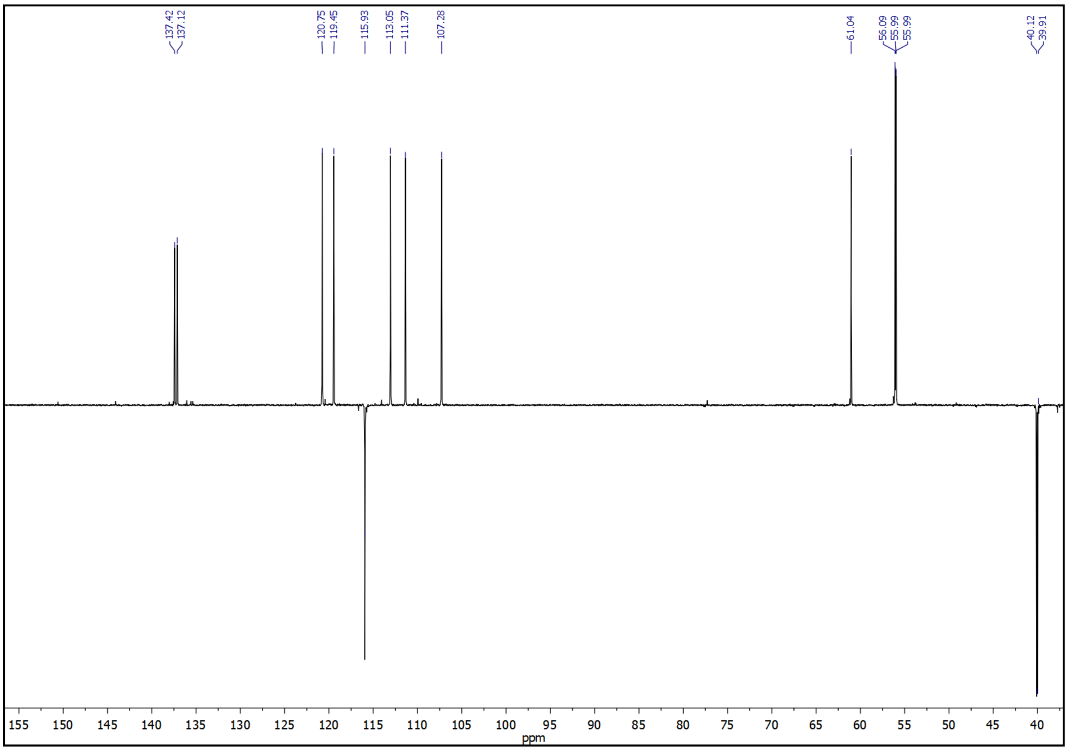

Figure S3. DEPT135 NMR spectrum from enlarged 40 to 155 ppm (δ, $CDCl_3$, 100 MHz) of **1**.

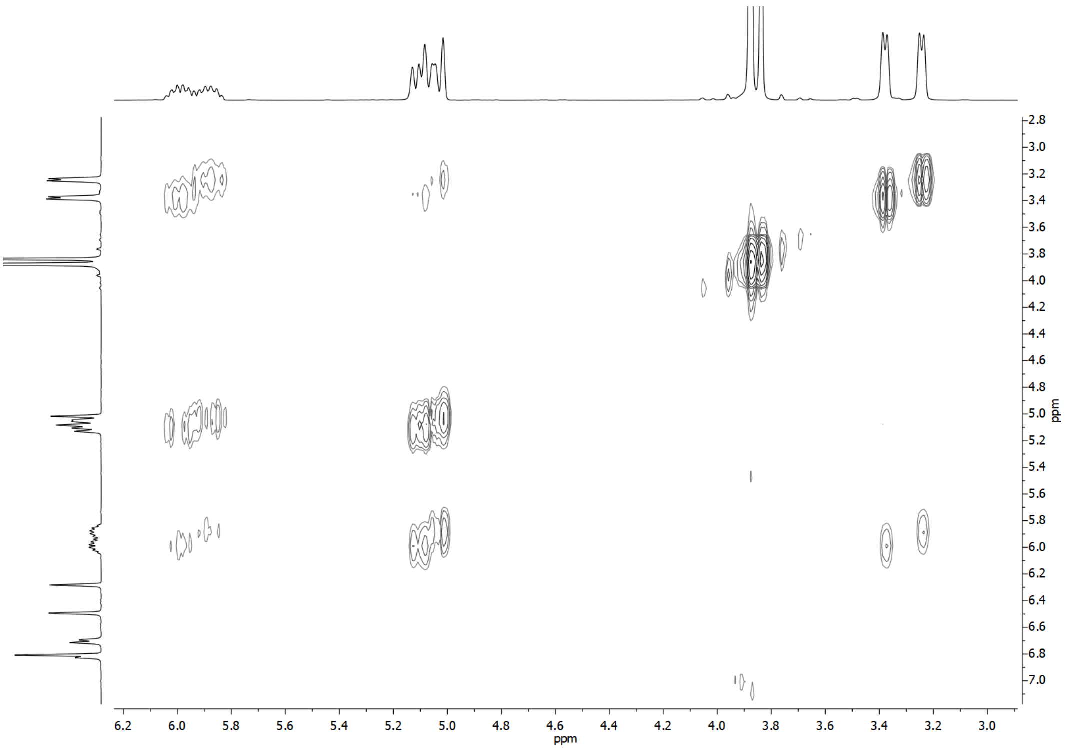

Figure S4. COSY contour map (δ, $CDCl_3$, 100 MHz) of **1**.

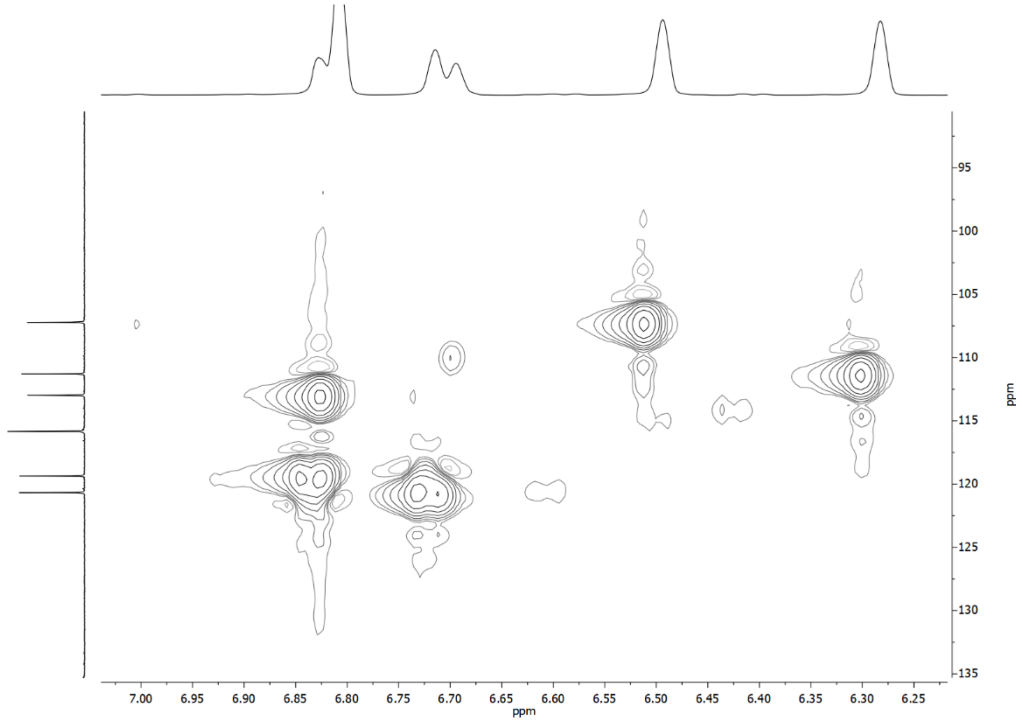

Figure S5. HSQC contour map (δ, $CDCl_3$, 400 and 100 MHz) of **1**.

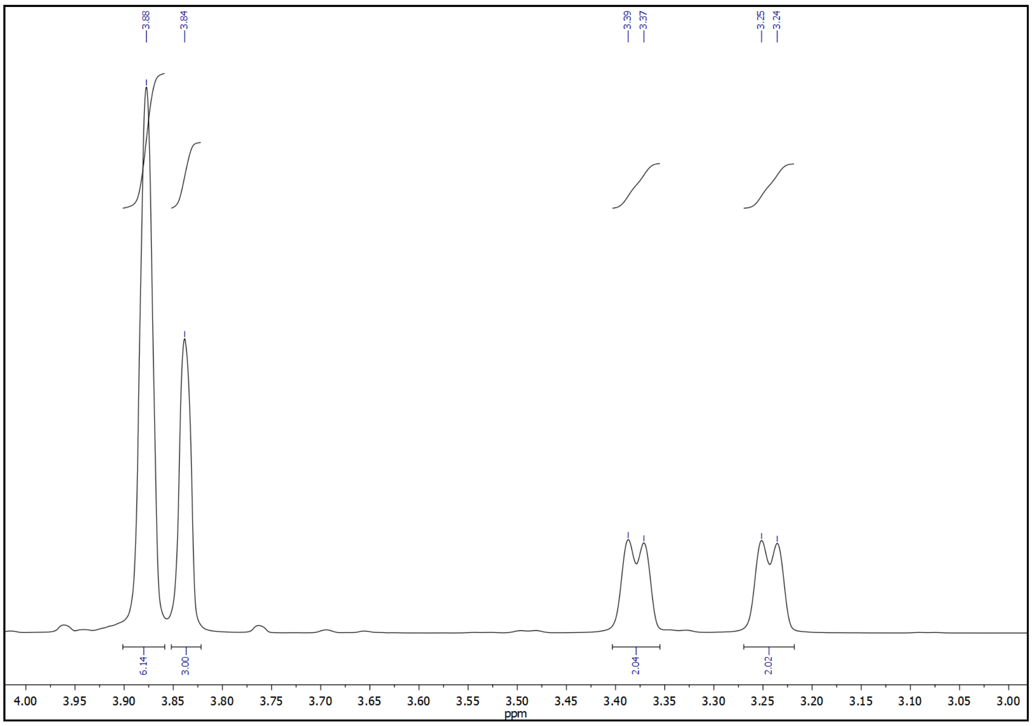

Figure S6. ^{1}H NMR spectrum (region from 3 to 4 ppm) (400 MHz, $CDCl_3$) of**1**.

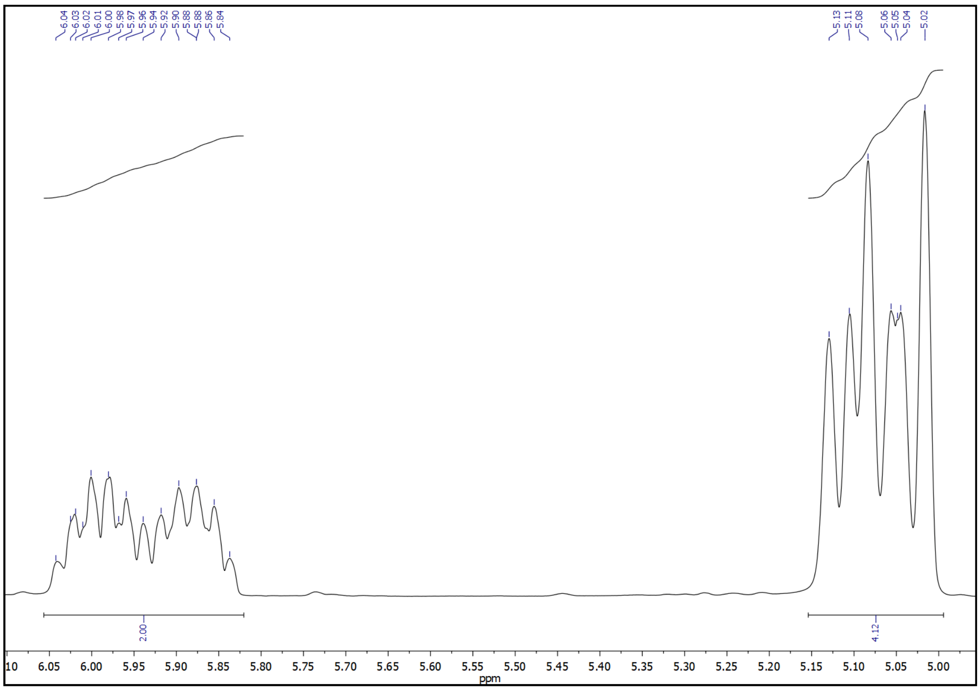

Figure S7. ^{1}H NMR spectrum (region from 5 to 6 ppm) (400 MHz, $CDCl_3$) of **1**.

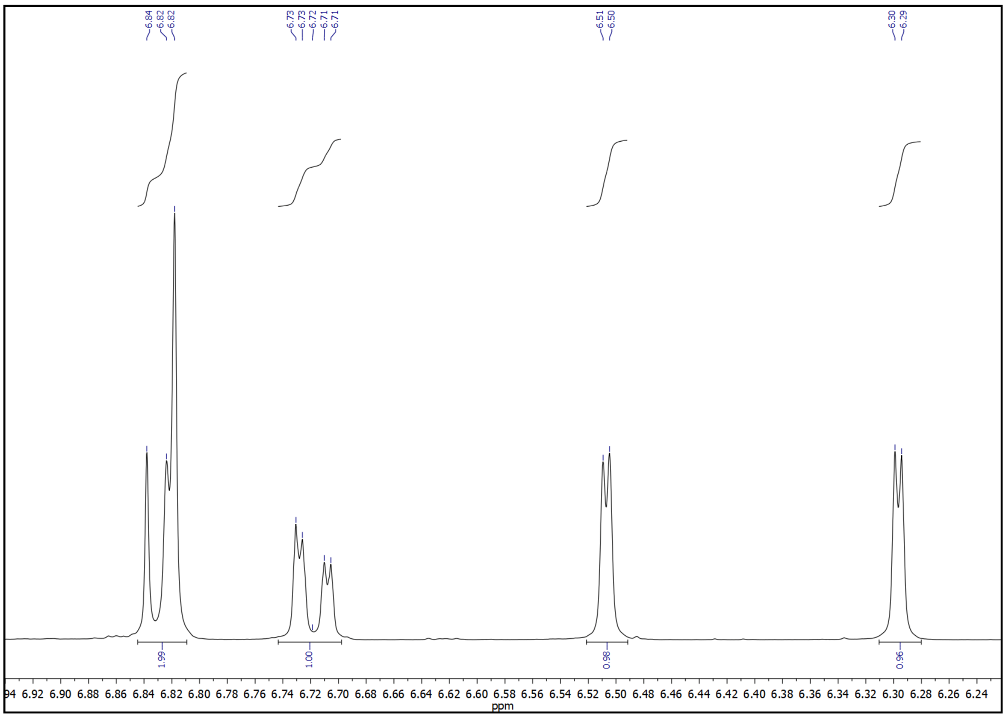

Figure S8. ^{1}H NMR spectrum (region from 6 to 7 ppm) (400 MHz, $CDCl_3$) of**1**.

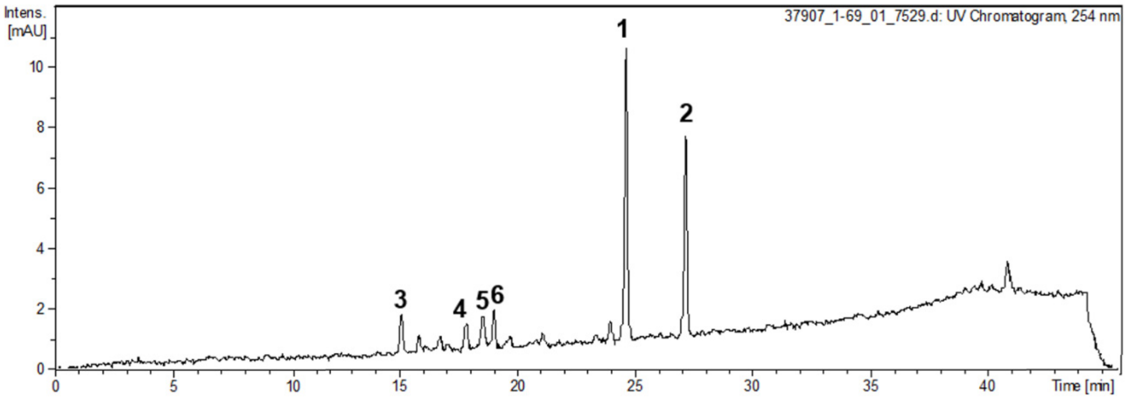

Figure S9. HPLC analysis of hexane extract from branches (HEB) of *E. paniculata*.

HPLC-ESI-HRMS conditions: mobile phase: H_2O (A) and MeOH HPLC grade (B); gradient: 50% B (0 min), 100% B (0–35 min), 100% B (35–40 min) 50% B (40–41 min) and 50% B (41–45 min). ionization parameters: 2 Bar nebulizer pressure, 8 L/min injection flow, secant gas at a temperature of 250 °C and 4.5 kV energy in the capillary.

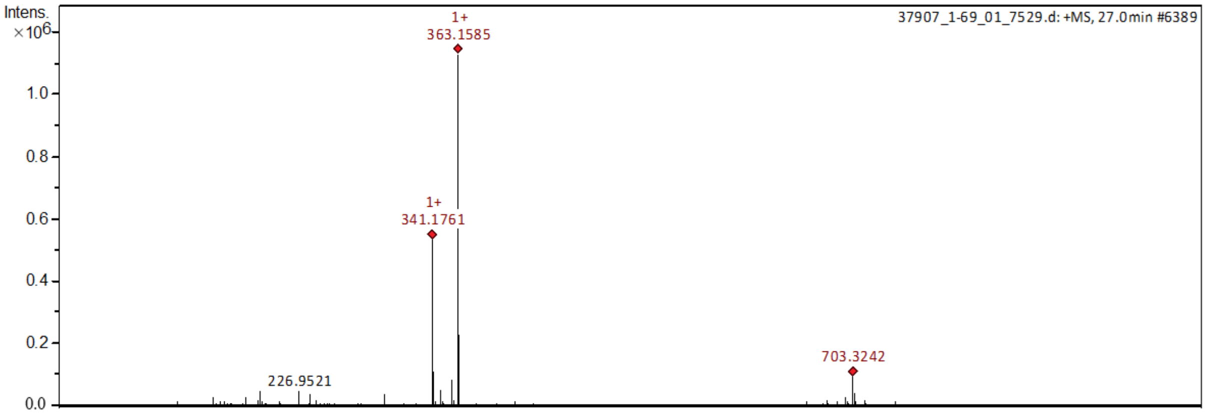

Figure S10. HPLC–ESI–MS spectrum (positive mode) of **1** at 27.0 min.

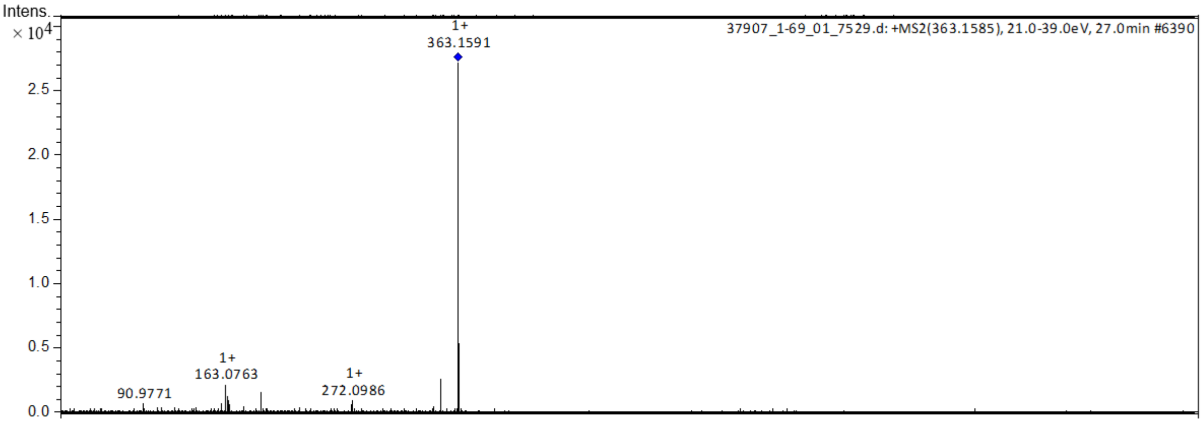

Figure S11. HPLC–ESI–MS/MS spectrum (positive mode) of **1** at 27.0 min.

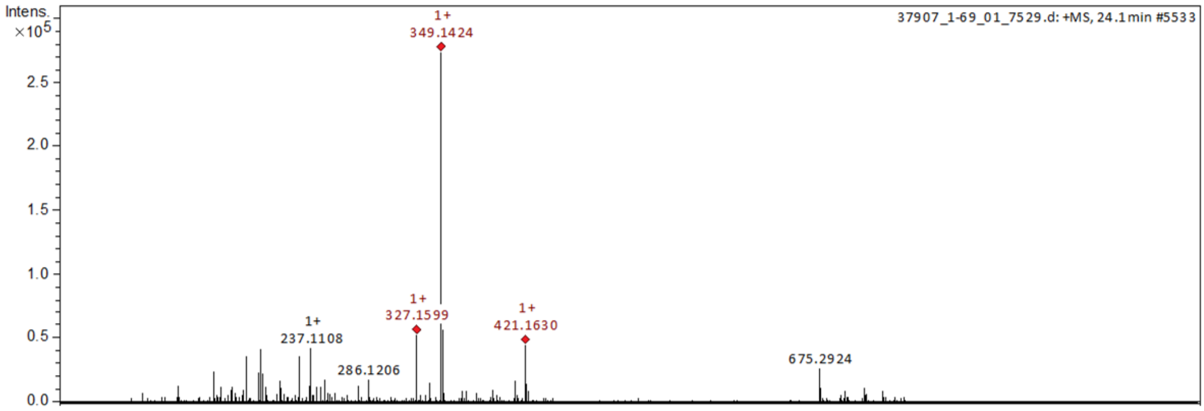

Figure S12. HPLC–ESI–MS spectrum (positive mode) of **2** at 24.1 min.

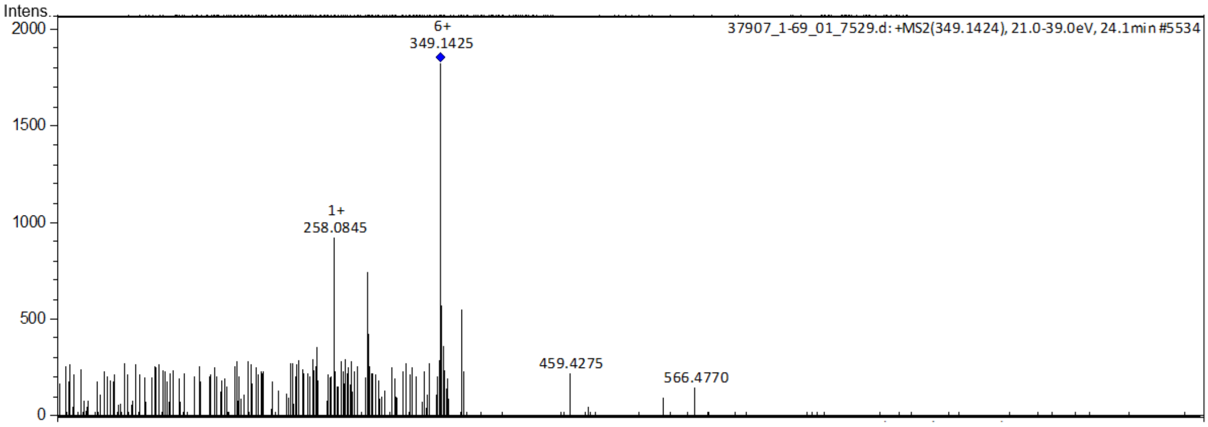

Figure S13. HPLC–ESI–MS/MS spectrum (positive mode) of **2** at 24.1 min.

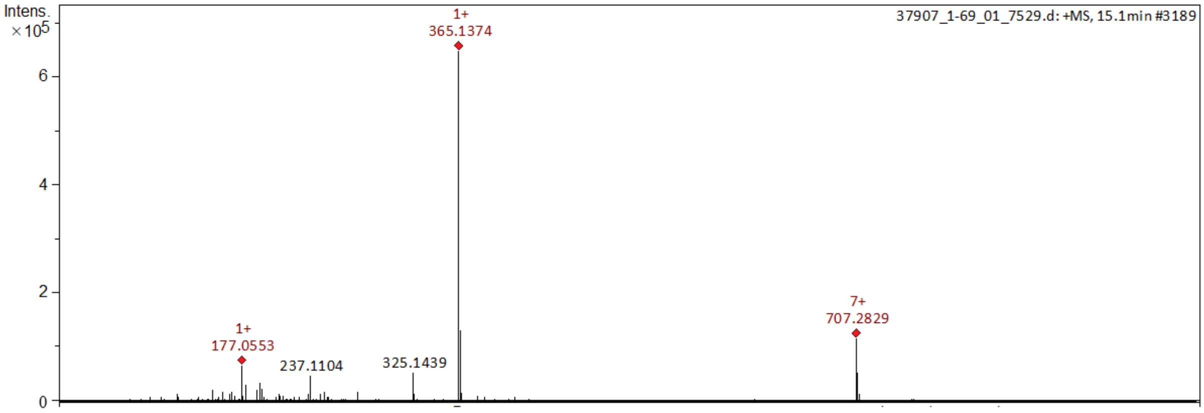

Figure S14. HPLC–ESI–MS spectrum (positive mode) of **3** at 15.1 min.

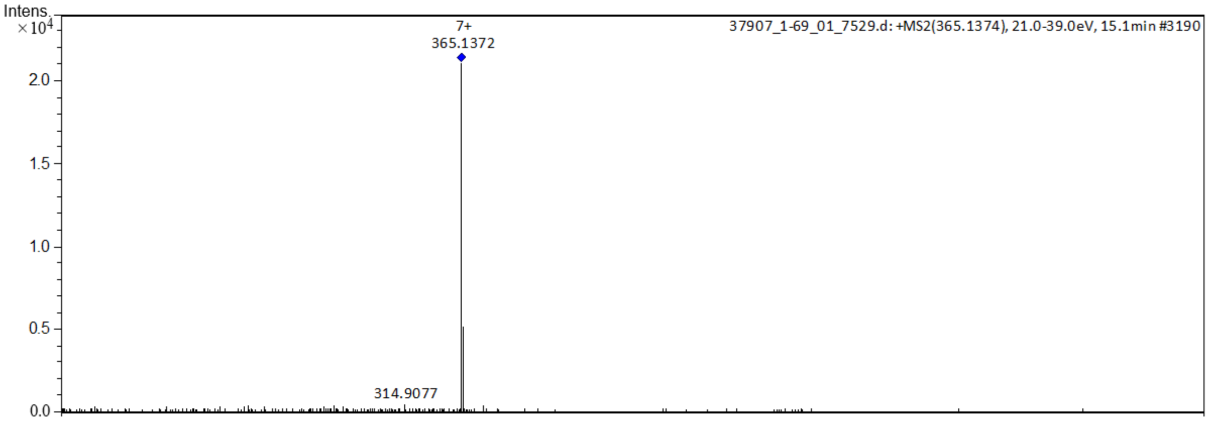

Figure S15. HPLC–ESI–MS/MS spectrum (positive mode) of **3** at 15.1 min.

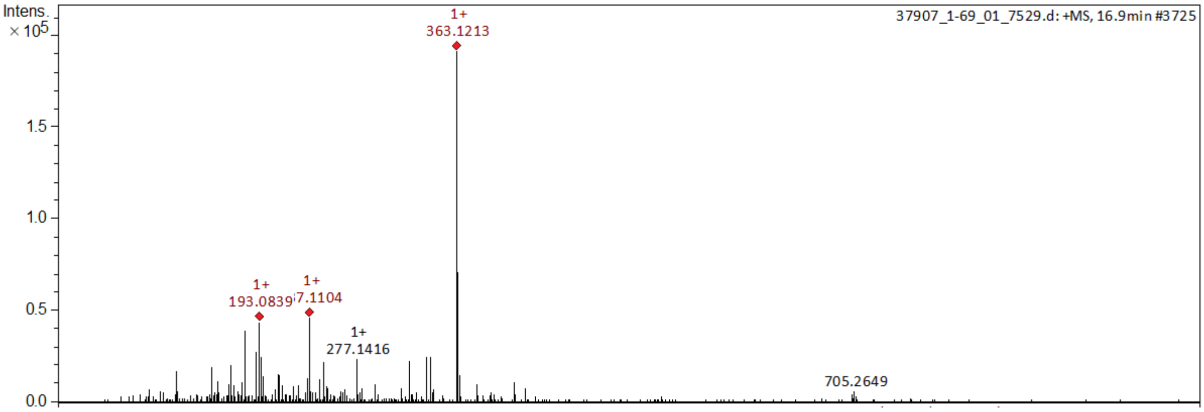

Figure S16. HPLC–ESI–MS spectrum (positive mode) of **4** at 16.9 min.

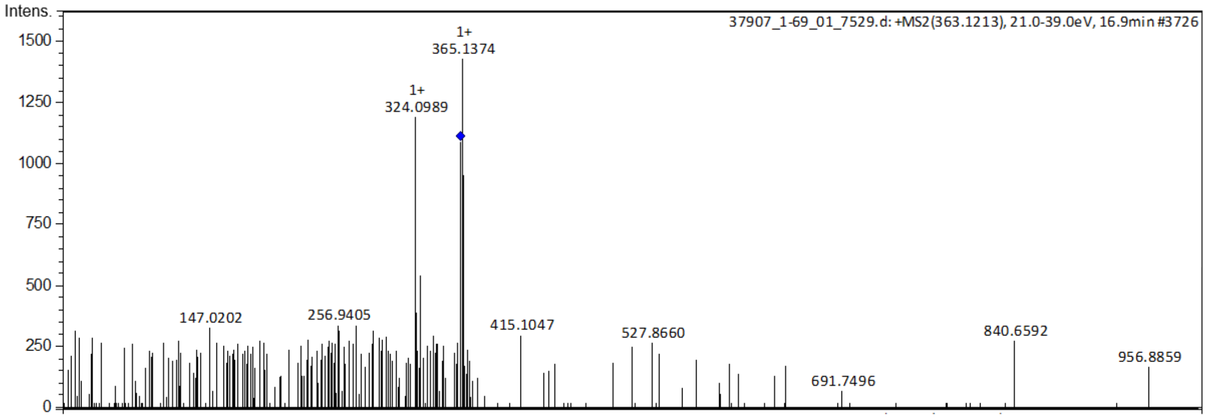

Figure S17. HPLC–ESI–MS/MS spectrum (positive mode) of **4** at 16.9 min.

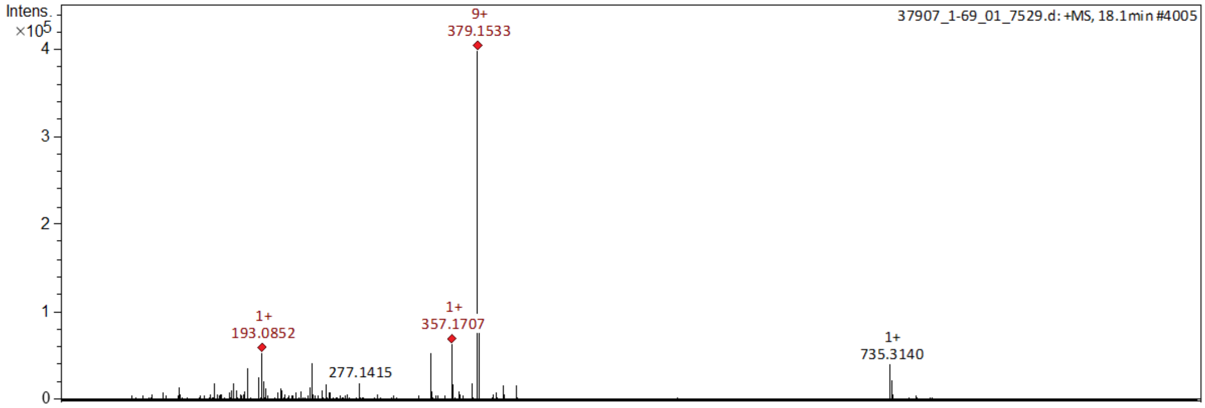

Figure S18. HPLC–ESI–MS spectrum (positive mode) of **5** at 18.1 min.

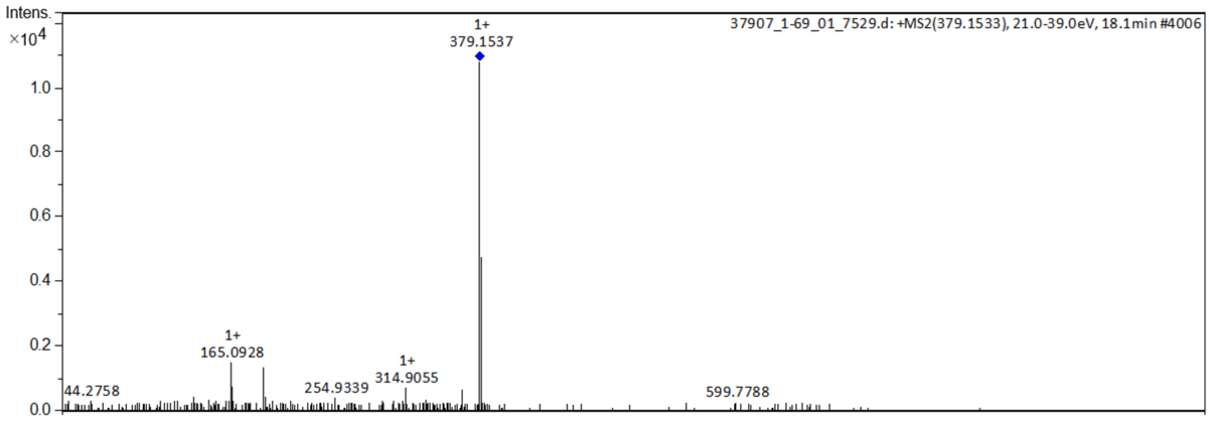

Figure S19. HPLC–ESI–MS/MS spectrum (positive mode) of **5** at 8.1 min.

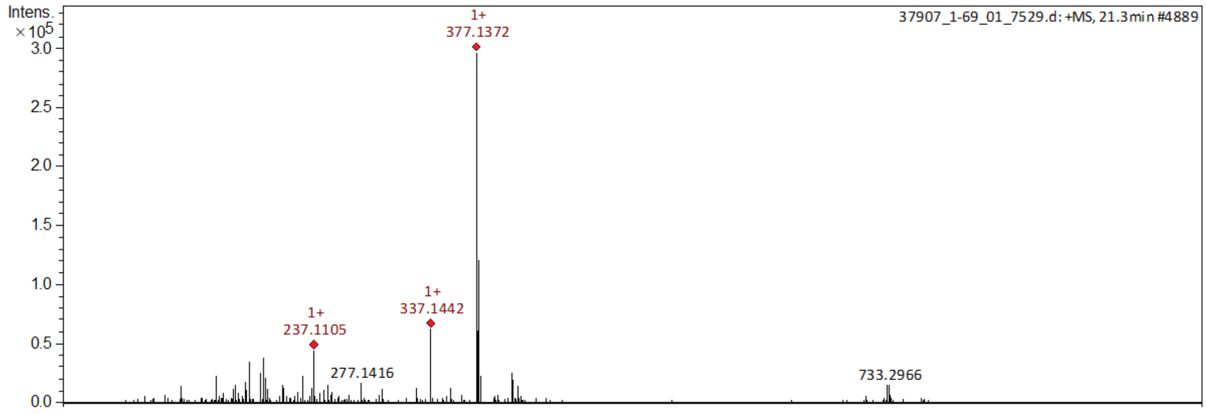

Figure S20. HPLC–ESI–MS spectrum (positive mode) of **6** at 21.3 min.

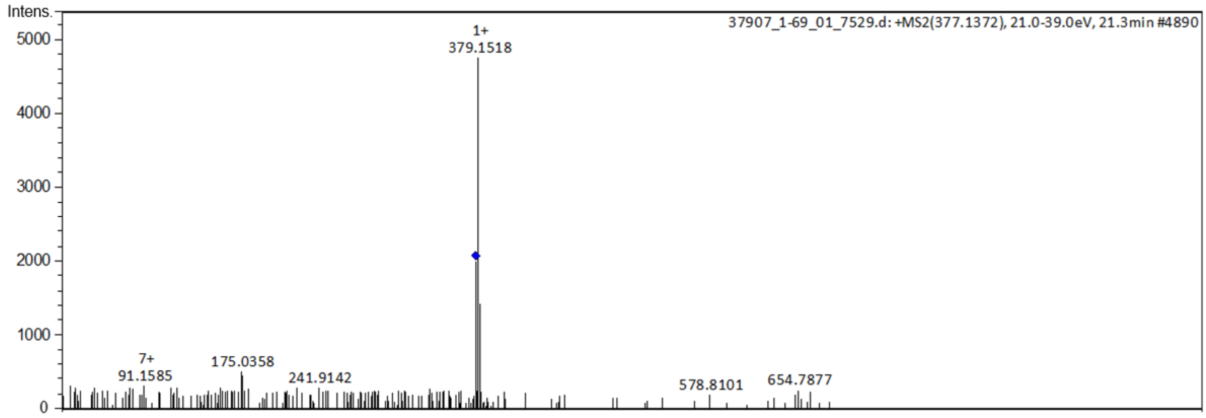

Figure S21. HPLC–ESI–MS/MS spectrum (positive mode) of **6** at 21.3 min.
